# Editorial: Perioperative organ dysfunction and protection in neonates and preterm infants

**DOI:** 10.3389/fped.2023.1271066

**Published:** 2023-10-05

**Authors:** John Zhong

**Affiliations:** Department of Anesthesiology and Pain Management, University of Texas Southwestern Medical Center, Dallas, TX, United States

**Keywords:** neonate, mortality, morbidity, hypothermia, acute kidney injury, hypertension

**Editorial on the Research Topic** Perioperative organ dysfunction and protection in neonates and preterm infants

## A global snapshot at potential ways to lower neonatal morbidity and mortality

Globally, there are approximately 2.3 million neonates who die in the first 28 days of their life from various causes. Prematurity secondary to maternal hypertensive disorder of pregnancy (HDP) remains a leading cause of very preterm birth. In this Research Topic in Frontiers in Pediatrics, (Ge et al.) report the rate of HDP among mothers of very preterm infants (VPI) in the Chinese Neonatal Network. This article perhaps shines new light on the effect of HPD on neonatal outcomes.

Not every country has the resources of China or Western countries. In this Research Topic in Frontiers in Pediatrics, (Abiy et al.) report a prospective cohort study on the incidence of death and its predictors among neonates admitted with sepsis to a referral hospital in northwest Ethiopia. They find low birth weight, prematurity, duration of labor longer than 24 h, not breastfeeding, having respiratory distress syndrome (RDS), and oxygen saturation lower than 90% as being the significant predictors of death among neonates admitted with sepsis. They diagnosed sepsis by questionaries rather than blood culture. Their findings are more relevant to low-income countries.

For some neonates, surgical intervention is required in the newborn period. One of the leading causes of perioperative complications is intra-operative hypothermia. Not only can hypothermia lead to catecholamine surges, increased metabolism, and vasoconstriction, but also leads to prolonged post-operative extubation times and surgical site infection. Despite the wide recognition of these detrimental effects, intra-operative hypothermia, defined as a temperature lower than 36°C, is still alarmingly high. In this Research Topic in Frontiers in Pediatrics, the retrospective observation study by Lai and colleagues (Zhao et al.) reports an 82.83% incidence of hypothermia intraoperatively despite the application of forced warm air mattresses and warmed intravenous fluid. It illustrates that there is still a lot of room for improvement. They further describe whether a cuffed endotracheal tube or low fresh gas flow technique were utilized and whether the setting of Operation Room temperature might have helped to pinpoint the causes for this high intra-operative hypothermia rate.

**Figure 1:**
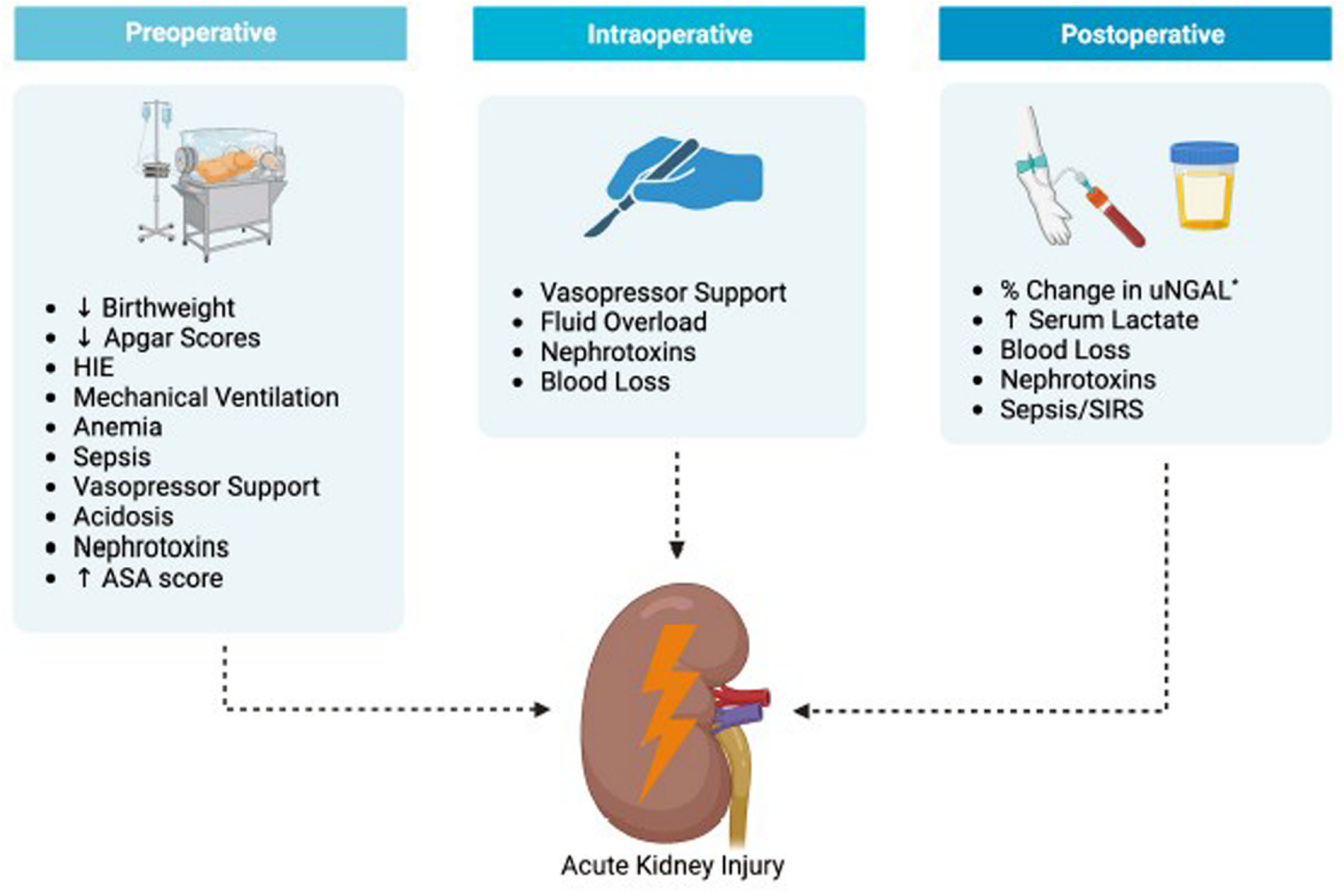
Risk factors associated with AKI development post abdominal surgery. HIE, hypoxic ischemic encephalopathy; uNGAL, urine neutrophil gelatinase-associated lipocalin; SIR, systematic inflammatory response syndrome *percent increase in uNGAL from pre- to 24 h postoperation.

Another common peri-operative complication, in particular for those neonates receiving intra-abdominal procedures, is acute kidney injury (AKI). In this Research Topic in Frontiers in Pediatrics, Duong and colleagues (Duong et al.) present a review of this topic. Figure 1 displays the associated risk factors for AKI divided into pre-operative, intra-operative, and post-operative stages. Equipped with this knowledge, clinicians need to maintain stable hemodynamics and euvolemia and avoid nephrotoxic medications such as gentamicin, vancomycin, and nonsteroid antiinflammation drugs (NSAIDs) to achieve prevention or mitigation of AKI.

From better-controlling hypertension during pregnancy and the early detection of sepsis among neonates, to maintaining normothermia intraoperatively and the prevention or mitigation of AKI, there seem to be a lot of opportunities for clinicians tasked with the care of neonates.

